# Fathers’ Cocaine Use and Parent–Child Feeding Interactions

**DOI:** 10.3390/jcm14041148

**Published:** 2025-02-10

**Authors:** Luca Cerniglia, Angelo Giovanni Icro Maremmani, Silvia Cimino

**Affiliations:** 1 Faculty of Psychology, International Telematic University Uninettuno, 00186 Rome, Italy; luca.cerniglia@uninettunouniversity.net; 2 Department of Health Sciences, UniCamillus—Saint Camillus International Medical University, 00131 Rome, Italy; angelogiovanniicro.maremmani@unicamillus.org; 3 Department of Clinical, Dynamic and Health Psychology, Sapienza, University of Rome, 00185 Rome, Italy

**Keywords:** father–child interactions, feeding, cocaine use, psychopathology

## Abstract

**Background:** Limited research has explored father–child interactions during feeding in dyads where fathers use cocaine, despite the critical role these interactions play in infant development. **Methods:** This study aimed to evaluate whether paternal cocaine use, psychopathology (measured via the SCL-90/R), and difficult child temperament (assessed using the QUIT) are linked to lower-quality father–child feeding interactions (evaluated through the SVIA) compared to dyads with non-substance-using fathers. **Results:** Father–child feeding interactions in the substance-using (SU) group were significantly poorer in quality than those in the non-substance-using (NSU) group. Fathers using cocaine displayed elevated SCL-90/R scores, particularly in hostility, anxiety, and depression. Maternal anxiety exacerbated interactional conflict during feeding. Furthermore, in the SU group, higher paternal psychoticism predicted lower-quality feeding interactions (as indicated by three SVIA subscales) but only when combined with higher levels of children’s Negative Emotionality. **Conclusions:** This study highlights the significant challenges faced by substance-using fathers in maintaining high-quality feeding interactions, emphasizing the detrimental impact of paternal psychopathology, maternal anxiety, and child temperament on caregiving dynamics.

## 1. Introduction

Parent–infant interactions are pivotal in shaping early developmental outcomes, laying the groundwork for children’s emotional, cognitive, and social growth [1]. Fathers, alongside mothers, contribute uniquely to these interactions, playing complementary roles in fostering a child’s ability to navigate their environment and regulate their emotions [2]. Research suggests that paternal engagement often centers on physical and stimulating activities, encouraging exploration, independence, and problem-solving, whereas maternal interactions typically emphasize emotional security and dyadic attunement [3,4]. The importance of paternal involvement is underscored by evidence linking active father–child engagement to better behavioral, emotional, and academic outcomes in children [5]. However, disruptions to paternal engagement—particularly due to substance use—may undermine these developmental benefits [6].

Fathers who use substances, such as cocaine, encounter distinct challenges in caregiving, often marked by withdrawal and emotional unavailability [7]. Unlike maternal substance use, which may result in heightened intrusiveness or over-control, paternal substance use frequently leads to disengaged or inconsistent parenting [8]. This withdrawal can manifest as reduced responsiveness to child cues, lower sensitivity during interactions, and a diminished ability to engage in sustained nurturing behaviors. Feeding interactions, in particular, require a high degree of sensitivity and responsiveness; paternal cocaine use may disrupt this dynamic, reducing the opportunities for fathers to reinforce emotional regulation and trust during such critical exchanges [9,10,11].

Moreover, paternal cocaine use may exacerbate pre-existing vulnerabilities, such as mental health challenges, that further impair parenting capacities [12]. Substance-using fathers are at greater risk of depression, anxiety, and emotional dysregulation, all of which can negatively influence the quality of their interactions with their children [13]. These psychiatric symptoms can result in fathers struggling to meet the demands of caregiving, particularly in high-stress contexts like feeding [14]. The interplay between substance use and mental health creates a compounding effect, where psychological distress both drives and is exacerbated by inadequate parenting, potentially establishing a negative feedback loop that affects the child’s developmental trajectory [15,16].

The effects of paternal cocaine use on child outcomes are not limited to impaired interactions. Cocaine use has been associated with broader environmental and genetic risks, including reduced caregiving stability and exposure to adverse home environments [17]. These factors can contribute to increased stress for children and the development of behavioral and emotional challenges. Furthermore, children of cocaine-using fathers often present with more difficult temperamental traits, such as heightened irritability and reduced ability to self-soothe [18,19]. Such traits can exacerbate the difficulties in dyadic regulation, further straining father–infant interactions and perpetuating cycles of maladaptive parenting and child distress [20].

Feeding contexts provide a particularly salient lens for studying father–infant interactions [21,22]. Unlike play or other caregiving activities, feeding necessitates sustained engagement and attunement to the infant’s needs and cues [23]. Fathers with cocaine use disorders may struggle to meet these demands due to impaired emotional regulation, reduced patience, or physical withdrawal symptoms [24]. Research on substance-using fathers highlights the risk of permissive or inconsistent parenting styles, where boundaries are either weak or unpredictably enforced [25]. These parenting patterns may undermine the critical regulatory benefits that infants derive from structured and predictable feeding interactions, hindering their ability to develop self-regulation and secure attachment [26].

Despite the growing recognition of fathers’ roles in early caregiving, much of the literature remains focused on mothers [27]. This gap limits our understanding of how paternal behaviors and characteristics influence child development, particularly in the context of substance use. Unlike mothers, fathers may experience unique stressors related to societal expectations of masculinity, financial responsibilities, and external pressures, which can compound the challenges of caregiving in the presence of substance use [28]. Addressing these gaps is crucial for developing interventions that support fathers in overcoming barriers to effective parenting.

This study exemplifies a replication effort, mirroring prior work on maternal cocaine use by focusing on fathers. Such symmetrical replication is useful, as it examines whether established findings hold true across different but related contexts, thereby enhancing the breadth and applicability of psychological theories. Furthermore, it underscores the need for parity in research focus, ensuring that both paternal and maternal roles in caregiving are adequately understood and integrated into developmental psychology. Replication of this nature not only tests the validity of earlier findings but also contributes to a more inclusive and holistic understanding of parent–child dynamics.

Replication studies are the cornerstone of scientific reliability and progression. In psychology, they serve to verify the robustness of findings by testing whether results from an original study can be consistently reproduced under similar or slightly varied conditions [29]. The replication of psychological research is particularly crucial given the complexity of human behavior and the myriad contextual factors that can influence outcomes. Successful replication reinforces confidence in the validity of a study’s conclusions, while failures to replicate provide opportunities to refine theoretical frameworks and methodological approaches [30].

In particular, this study aims to investigate the effects of paternal cocaine use, psychopathology, and child temperament on father–infant feeding interactions. We hypothesize that cocaine use will be associated with reduced sensitivity, emotional availability, and consistency during feeding interactions. Additionally, we propose that paternal psychopathology and child temperament will be associated with cocaine use and interaction quality. Finally, we will explore the potential interactive effects of cocaine use and psychopathology, identifying how these factors jointly influence father–infant interaction.

## 2. Materials and Methods

### 2.1. Sample

Fathers and children participating in this study were selected from a larger research project [31] aimed at promoting offspring psychological well-being in the general population. This larger study involved administering anamnestic questionnaires, which included questions on alcohol and substance use. From this dataset, a subgroup of fathers who reported cocaine use was identified (fathers and their children; substance use—SU, N = 122). A matched control group of non-substance-using fathers was selected based on age and socioeconomic status (SES) (non-substance use—NSU).

The fathers in the sample had an average age of 30.12 years (SD = 3.44), and their children had an average age of 2.26 years (SD = 0.52). The sample included 48% female children. All fathers were biological parents, serving as primary caregivers with daily contact with their children. Most families were intact (94%), and all participants were Caucasian. Despite research linking substance use to lower SES, the fathers in this study primarily belonged to middle-to-upper socioeconomic groups, with household incomes between EUR 35,000 and EUR 45,000 per year. No statistically significant differences were found between the SU and NSU groups in demographic characteristics (χ^2^; *p* < 0.001). Exclusion criteria for both groups included (a) a psychiatric diagnosis or (b) current engagement in medical or psychological treatment. All participants signed written informed consent forms, and the study was approved by the Ethical Committee before its commencement (Protocol N. 13/2022). Father–child pairs were observed in their homes during a 20 min midday lunch, which was video-recorded. Trained psychologists conducted the recordings and applied a validated observational protocol. Two independent raters analyzed the recordings using a coding manual and software to compute scores on specific subscales. The observations adhered to the Scala di Valutazione dell’Interazione Alimentare (SVIA), an Italian observational tool designed to evaluate parent–child interactions during feeding [32]. This tool was selected for its focus on feeding-specific dynamics, distinguishing it from other general observational instruments. Fathers completed two additional assessments at home: the Symptom Checklist-90-Revised (SCL-90-R; [33]), which measured psychological distress, and the Italian Questionnaires on Temperament (QUIT; [34]), which assessed child temperament.

### 2.2. Assessment

#### Psychological Symptoms of Fathers

The SCL-90-R is a 90-item self-report inventory designed to measure psychological symptoms and distress. Participants rate the degree to which they experienced symptoms in the past week on a Likert scale from 0 (not at all) to 4 (extremely). It includes nine subscales (e.g., depression, anxiety, hostility; item examples, respectively: feeling low in energy or slowed down; trembling; temper outbursts that you could not control) and three global indices of distress. Previous research has demonstrated its high internal consistency across different populations (in this study, α = 0.85).

The QUIT is a parent-report questionnaire validated for Italian populations across various age groups. It evaluates six dimensions of child temperament, such as motor activity, attentional capacity, and emotionality (item examples, respectively: when he/she plays, he/she is constantly moving; when he/she does a task, he/she looks around; as soon as you speak to him/her, he/she puts on a smiling expression), using a Likert scale from 1 (almost never) to 6 (almost always). Fathers completed the QUIT independently, with high agreement typically observed between parental reports (in this study, Cronbach’s α = 0.88).

The SVIA Feeding Scale is an observational tool that evaluates dyadic feeding interactions through 41 items rated on a Likert scale from 0 (none) to 3 (many). Four subscales assess specific dimensions: (1) Affective State of the Parent： Higher scores indicate challenges in displaying positive emotions and a higher prevalence of negative emotions such as sadness or frustration. (2) Interactional Conflict measures the intensity and frequency of conflict within the dyad during feeding. (3) Food Refusal Behaviors of the Child examines emotional and behavioral feeding-related patterns in the child. (4) Affective State of the Dyad reflects difficulties in establishing supportive interactions, often manifesting as distress or oppositional behaviors in the child and challenges in the caregiver’s responsiveness. The SVIA has demonstrated strong psychometric properties, including discriminant validity (classification accuracy: 82–92%) and interrater reliability (κ = 0.82–0.92). In this study, two specifically trained coders (who had obtained reliability from the authors of the measure) scored the feeding interactions with an agreement of 91%.

### 2.3. Data Analysis

Initial data screening revealed minimal missing data (<3% for each measure), which was addressed through multiple imputations using SPSS (Version 25.0). To analyze differences in feeding interactions between the SU and NSU groups, multivariate analyses of variance (MANOVAs) were performed on the SVIA subscales. Significant findings were followed by univariate analyses with Duncan tests and Bonferroni corrections for post hoc comparisons. Fathers’ psychological symptom statuses were compared between groups using MANOVAs based on SCL-90-R indices (transformed to square root when normality was violated). The criterion variables were group (SU vs. NSU), and the dependent variables were scores from the psychological and observational measures. Finally, two hierarchical regression analyses were conducted to examine the influence of specific SCL-90-R subscales and children’s temperament (QUIT dimensions) on feeding interaction scores. The child’s sex was not found to have a significant impact on any variable. All analyses were conducted using SPSS (Version 25.0).

## 3. Results

### 3.1. Quality of Father–Child Interactions During Feeding

Multivariate analyses revealed significantly higher SVIA subscale scores in the SU group compared to the NSU group (*p* < 0.01). Approximately 15% (N = 28) of the SU dyads exceeded clinical cutoffs (>54) on the SVIA, indicating significant challenges in father–child interactions (Table 1).

### 3.2. Assessment of Paternal Psychopathological Risk

The analysis of psychological symptoms revealed that fathers in the SU group scored significantly higher than those in the NSU group on the subscales of hostility, depression, and anxiety. Furthermore, fathers in the SU group showed elevated scores on the Global Severity Index of the SCL-90-R (*p* < 0.001), as detailed in Table 2. These findings suggest that paternal substance use is associated with heightened levels of psychological distress.

Additionally, 30 fathers (17%) in the SU group scored above the clinical cutoffs on the SCL-90-R, indicating potential psychological concerns that may warrant further evaluation. This elevated psychopathological risk highlights the importance of considering both substance use and mental health factors when assessing the overall caregiving environment in father–child dyads.

### 3.3. Quality of Father–Child Interactions During Feeding Considering Psychopathological Risk and Child Temperament

Regression analyses were performed for the two groups to investigate the impact of SCL-90-R subscales and temperament subscales on the four relational dimensions of the SVIA in father–child interactions (Table 3). In the NSU group, the findings showed that higher maternal psychoticism scores were associated with more negative effects in the Father’s Affective State (*p* < 0.05). Furthermore, higher scores for children’s Negative Emotionality, as measured by the QUIT, were linked to more maladaptive father–infant interactions in the Dyad’s Affective State during feeding (*p* < 0.001).

In the group of fathers who used cocaine, the regression analyses indicated that elevated paternal depression scores were associated with higher scores (reflecting poorer quality) on three SVIA subscales—Interactional Conflict, Food Refusal by the Child, and Dyad’s Affective State—but only when paired with higher scores of children’s Negative Emotionality (*p* < 0.05).

## 4. Discussion

This study underscores the profound challenges that substance-using (SU) fathers face in fostering high-quality feeding interactions with their children, highlighting a range of difficulties that disrupt the emotional and relational fabric of caregiving [35]. Fathers in the SU group exhibited significantly higher scores on all subscales of the SVIA compared to non-substance-using (NSU) fathers, reflecting notable impairments in relational quality. Furthermore, 15% of SU father–child dyads exceeded clinical cutoffs, a threshold indicating interactional difficulties of clinical significance. These disruptions were particularly evident in feeding-specific contexts, where maladaptive patterns emerged in areas such as Food Refusal and the Affective State of the Dyad. These results align with prior research on parental substance use, which consistently highlights the erosion of caregiving sensitivity and responsiveness as central outcomes of substance-related impairments [36].

A critical factor influencing these difficulties lies in the dual impact of paternal psychopathological risks and child temperament. Depression and Negative Emotionality in children emerged as key contributors to maladaptive interactions, suggesting a bidirectional relationship between the emotional states of fathers and their children [37,38]. This is consistent with theories of dyadic regulation, which emphasize the interplay of parent and child affective states in shaping relational dynamics [39]. Feeding, a caregiving activity requiring sustained emotional availability and attunement to child cues, appears particularly susceptible to the challenges posed by paternal substance use [40]. SU fathers may struggle to maintain the patience, sensitivity, and consistency necessary for positive feeding interactions, leading to disruptions in the regulatory benefits these interactions typically provide for children.

The pronounced psychopathological risks in SU fathers further compound these challenges. Fathers in the SU group scored significantly higher on measures of depression, anxiety, and hostility, with 17% surpassing clinical cutoffs on the SCL-90-R. The emergence of hostility as a distinguishing characteristic in fathers, absent in maternal findings from similar studies [41], suggests a gendered dimension to the expression of psychopathological risks in substance-using parents. Hostility, often an externalized symptom of psychological distress, may manifest in interactions as irritability or disengagement, further undermining relational quality [42]. These patterns are particularly concerning given the critical role that fathers play in promoting exploration, problem-solving, and emotional regulation in children [43]. Without intervention, these impairments could disrupt developmental trajectories, hindering children’s capacity to build secure attachments and effective self-regulation mechanisms.

Notably, the findings also illuminate how child temperament interacts with paternal psychopathology to shape relational dynamics. Negative Emotionality in children—a temperament trait marked by heightened reactivity and difficulty in self-soothing—was a significant predictor of feeding challenges in SU dyads. This trait, when combined with paternal depression, exacerbated maladaptive interactions in key relational domains. These results highlight the reciprocal nature of parent–child dynamics, where child temperament not only influences but is also shaped by the quality of caregiving [44]. The compounded effect of these factors underscores the need for comprehensive interventions that address both paternal mental health and child behavioral support.

This study replicated a prior investigation focusing on mothers, enabling a comparison of specific patterns in caregiving under substance use conditions. While both SU fathers and mothers demonstrated significant impairments in parent–child feeding interactions, several differences were observed. First, a higher proportion of mother–child dyads (24%) exceeded clinical cutoffs on the SVIA compared to father–child dyads (15%), indicating that maternal interactions may exhibit greater observable difficulties during feeding. This aligns with research suggesting that maternal substance use more broadly impacts caregiving dimensions beyond feeding-specific interactions [45]. Second, while both groups of SU parents exhibited elevated depression and anxiety, fathers showed higher hostility, whereas mothers demonstrated greater Interpersonal Sensitivity. These differences reflect potentially different manifestations of substance-related psychological distress, with fathers displaying externalized symptoms and mothers showing heightened relational sensitivity [46]. Third, child Negative Emotionality interacted with parental psychopathology in both fathers and mothers, influencing feeding interactions. However, the effect was more pervasive in mothers, impacting three SVIA subscales (Interactional Conflict, Food Refusal, Affective State of the Dyad) compared to two in fathers. This suggests that maternal caregiving may be more sensitive to child temperament, potentially due to differences in caregiving roles [34].

This study addresses a significant gap in the literature by examining paternal caregiving under substance use conditions, a field historically dominated by research on mothers [15] and has several strengths. By replicating a prior study on maternal substance use, this research enhances the reliability of findings and offers a comparative lens to understand gender-specific dynamics in caregiving [31]. The use of validated observational tools (SVIA) and standardized psychopathological assessments (SCL-90-R) ensures robust data collection and analysis. However, this study also has a number of limitations. The study focused on Caucasian, middle-to-upper socioeconomic families, limiting generalizability to more diverse populations or lower socioeconomic groups [37]. Moreover, the findings are based on single time-point assessments, which preclude the examination of longitudinal changes in paternal caregiving behaviors under substance use conditions. Finally, while feeding is a critical context for parent–child bonding, other caregiving domains, such as play or bedtime routines, were not examined, potentially overlooking broader relational dynamics.

## 5. Conclusions

This study sheds light on the unique challenges faced by SU fathers in parent–child feeding interactions, contributing to the growing recognition of paternal roles in early caregiving. By comparing these findings with maternal studies, it highlights gender-specific vulnerabilities and strengths, calling for tailored interventions that address both parental psychopathology and child temperament. Future research should explore longitudinal trajectories and include more diverse populations to enhance the applicability of findings. 

## Figures and Tables

**Table 1:** Means (SDs), F, and *p* values of SU group and NSU group scores on SVIA subscales.

	SU Group	NSU Group	Fisher *F* Test	*p*
Affective State of the Father	6.13 (2.65)	14.15 (3.52)	*F*(1, 243) = 92.56	<0.001
Interactional Conflict	3.54 (2.61)	11.52 (4.51)	*F*(1, 243) = 73.62	<0.001
Food Refusal of the Child	2.29 (1.42)	7.46 (3.23)	*F*(1, 243) = 67.43	<0.001
Affective State of the Dyad	2.90 (1.39)	7.98 (2.62)	*F*(1, 243) = 60.24	<0.001

**Table 2:** Means (SDs), F, and *p* values of mothers’ and fathers’ scores on SCL-90-R subscales.

	SU Group	NSU Group	Fisher *F* Test	*p*
Somatization	0.43 (0.56)	0.45 (0.61)	*F*(1, 243) = 26.27	n.s.
Obsessive Compulsive	0.34 (0.54)	0.39 (0.46)	*F*(1, 243) = 29.56	n.s
Interpersonal Sensitivity	0.43 (0.43)	0.51 (0.35)	*F*(1, 243) = 28.52	n.s
Depression	0.31 (0.34)	0.71 (0.45)	*F*(1, 243) = 34.72	<0.001
Anxiety	0.53 (0.62)	0.86 (0.68)	*F*(1, 243) = 29.14	<0.001
Hostility	0.47 (0.61)	0.71 (0.56)	*F*(1, 243) = 31.57	<0.001
Phobic Anxiety	0.44 (0.64)	0.51 (0.56)	*F*(1, 243) = 28.42	n.s.
Paranoid Ideation	0.24 (0.23)	0.26 (0.65)	*F*(1, 243) = 26.59	n.s.
Psycoticism	0.53 (0.27)	0.58 (0.43)	*F*(1, 243) = 29.56	n.s.
Global Severity Index	0.44 (0.37)	0.91 (0.53)	*F*(1, 243) = 32.68	<0.001

**Table 3:** Results and values of the regression analyses.

SVIA	QUIT—Negative Emotionality				SCL-90-R—Psychoticism			
**NSU Group**	**R^2^**	**ß**	**t**	***p***	**R^2^**	**ß**	**t**	***p***
Affective State of the Mother	n.s.	n.s.	n.s.	n.s.	0.242	0.349	2.614	0.042 *
Affective State of the Dyad	0.001	0.513	2.687	0.001 **	n.s.	n.s.	n.s.	n.s.
	**SCL-90-R—Depression**							
**SU Group**	**R^2^**		**ß**		**t**		***p***	
Interactional Conflict•Negative Emotionality	0.143		0.391		3.453		0.001 *	
Food Refusal of the Child•Negative Emotionality	0.071		0.252		2.541		0.031 *	
Affective State of the Dyad•Negative Emotionality	0.159		0.325		3.246		0.002 *	

Note: The subscales that are not shown in the table are not statistically significant. * *p* < 0.05; ** *p* < 0.01; n.s. = non-significant. • Association with.

## Data Availability

Data are available by reasonable request to the authors.
